# Oligo-FISH-Based Analysis of the Mechanisms Underlying Chromosome Number Variation in *Saccharum spontaneum*

**DOI:** 10.3390/ijms26051958

**Published:** 2025-02-24

**Authors:** Maoyong Ran, Bo Yu, Chunxia Cheng, Xueting Li, Yirong Guo, Liping Zhao, Fenggang Zan, Xiuqin Lin, Xiao Hou, Yong Zhao, Jiayong Liu, Zuhu Deng

**Affiliations:** 1 National Engineering Research Center for Sugarcane, Fujian Agriculture and Forestry University, Fuzhou 350002, China; 2 National Key Laboratory for Biological Breeding of Tropical Crops, Kunming 650205, China; 3 Yunnan Key Laboratory of Sugarcane Genetic Improvement, Sugarcane Research Institute, Yunnan Academy of Agricultural Sciences, Kaiyuan 661699, China

**Keywords:** *S. spontaneum*, Oligo-FISH, chromosome karyotype, chromosome inheritance

## Abstract

Interspecific hybridization serves as a crucial strategy for innovating sugarcane germplasms. Currently, nearly all modern sugarcane varieties that incorporate genetic material are derived from *Saccharum spontaneum*. The number of chromosomes in *S. spontaneum* ranges from 40 to 128, contributing significantly to the diversity of its genetic resources. However, the genetic mechanisms driving chromosome number variation in *S. spontaneum* remain to be fully elucidated. Here, oligonucleotide fluorescence in situ hybridization (Oligo-FISH) was conducted to identify individual chromosomes and explore chromosome transmission during the intraspecific hybridization of *S. spontaneum*. The results indicate that from the progenies generated from *S. spontaneum* Yunnan2017-22 (2*n* = 8*x* = 64) and Yunnan82-1 (2*n* = 8*x* = 64) emerged two distinct karyotypes, 2*n* = 12*x* = 96 (A1) and 2*n* = 8*x* = 64 (A2, A33-1, A18). This implies that the chromosome inheritances were 2*n* + *n* and *n* + *n* in the progenies. However, self-pollinated samples of A1 (2*n* = 12*x* = 96) produced normal offspring C1 (2*n* = 94) and C2 (2*n* = 96). The 2*n* + *n* inheritance pattern did not continue. In another cross, the progenies derived from *S. spontaneum* Yunnan2017-41 (2*n* = 8*x* = 64) and Yunnan8 (2*n* = 10*x* = 80) carried a karyotype of 2*n* = 9*x* = 72, with *n* + *n* inheritance mode. These findings highlight the existence of two chromosome inheritance modes, 2*n* + *n* and *n* + *n*, in the context of the intraspecific hybridization of *S. spontaneum*. Additionally, hybridization between different ploidy *S. spontaneum* was also accompanied by chromosomal translocations (A1, A2, A18, A18) and loss (A2, A33-1, AA-4, and C2) that further resulted in the complexity of the *S. spontaneum* genome. Together, these findings highlight diverse chromosome inheritance in *S. spontaneum* hybridization, and provide a theoretical foundation for the further utilization of *S. spontaneum* germplasm in sugarcane breeding.

## 1. Introduction

Sugarcane is a crucial crop for both sugar and bioenergy production worldwide. It accounts for approximately 80% of global sugar production, which is more than four times the output of beet sugar production [1]. In global sugarcane production, 81% is utilized for sugar production, while 19% is dedicated to bioethanol production [2]. As societal development progresses, the emphasis on sustainable energy production has grown, prompting major sugarcane-producing countries such as Brazil and India to increasingly shift from sugar production to sugarcane ethanol production [3]. Additionally, sugarcane by-products, including bagasse and cane tips, serve as valuable feed resources [4]. Sugarcane is one of the most significant crops that directly influences global sugar security. Germplasm innovation is one of the most important ways of increasing the yield of sugarcane breeding. *Saccharum spontaneum* is one of the most successful wild species utilized in sugarcane breeding, and plays an essential role in improving sugarcane resistance.

*S. spontaneum* is a perennial herb belonging to the genus *Saccharum* within Poaceae. This species reproduces both sexually and asexually and exhibits considerable intraspecific variation, with chromosome numbers widely ranging from 2*n* = 40 to 2*n* = 128 [5,6,7]. Over the past century, *S. spontaneum* has consistently served as a vital genetic resource for traits such as disease resistance, drought tolerance, and robust perennial root development in sugarcane breeding [8]. It is regarded as a significant parent in the distant hybridization of sugarcane and represents a wild germplasm resource that holds promise for further development and utilization within the *Saccharum* complex [9].

As of December 2020, there were 961 preserved germplasm resources of *S. spontaneum* in the National Sugarcane Germplasm Resources Nursery of China [10] that were widely distributed in nature and encompassed a rich diversity of types. However, their utilization remains extremely limited. According to statistics on the use of *S. spontaneum* among 20 backbone parents used in sugarcane hybrid breeding in mainland China, Liu et al. reported that only 6 utilized clones were identified [11]. These clones primarily include *S. spontaneum* from India, Java (Indonesia), and specific regions in China such as Yachen (Hainan) and Taiwan Province. The limited development and utilization of *S. spontaneum* in sugarcane breeding can be attributed to the long-term consumption and labor-intensive nature of the work involved. Despite the abundance of genetic resources available, only a few clones have been successfully applied in sugarcane breeding. In the 1910s, Jeswiet pioneered noblization breeding in sugarcane, which utilized *S. spontaneum* for developing the first renowned sugarcane cultivar, POJ2878, known as the “King of Sugarcane” [12]. The latest generation of elite sugarcane, YZ08-1609, is also a descendant of *S. spontaneum* originating from Yacheng [13]. This highlights that the utilization of *S. spontaneum* has a significant contribution to sugarcane breeding.

In traditional breeding methods, *S. spontaneum* is utilized as the male and *Saccharum officinarum* serves as the female in the process of hybridization and successive backcrosses [14]. The presence of the *2n*+ *n* genetic mechanism during selection allows for the retention and enhancement of high-sugar and high-yield traits from *S*. *officinarum* while simultaneously integrating the stress-resistant genes of *S. spontaneum* through chromosomal recombination [15]. This process results in the development of sugarcane varieties characterized by high sugar content and strong stress resistance [16]. *S. spontaneum* plays a crucial role in sugarcane breeding as a source of resistance genes. However, its undesirable traits, such as fine stems and low sugar content, extend the breeding cycle. Therefore, it is necessary to enhance resistance and to produce thicker stems and higher sugar contents of *S. spontaneum* through intraspecific cross-breeding. Previous studies have demonstrated that *S. spontaneum* possesses a very complex chromosome composition, characterized not only by an extensive range of chromosome numbers but also by three different basic chromosome numbers, *x* = 10, *x* = 9, and *x* = 8. Piperidis et al. was the first to propose an intermediate chromosome with *x* = 9 [17], indicating that the transition from *x* = 10 to *x* = 8 did not occur overnight during the long course of evolution. Meng et al. reported that this evolutionary process involves the fracture and fusion of two chromosomes [18,19]. Zhang et al. provided genomic insights into this phenomenon, confirming that the transition involved a reduction from *x* = 10 to *x* = 8. Furthermore, a comparative analysis of the genome of Np-X with the tetraploid AP85-441, developed from the anther clone of octoploid *S. spontaneum* SES208, substantiated that *S. spontaneum* with *x* = 10 is the original ancestor of *x* = 8 and *x* = 9 [5,7]. However, the chromosome inheritance of *S. spontaneum* hybridization is still unclear.

Fluorescence in situ hybridization (FISH) is a rapidly developed technology that has gained prominence in recent years [20]. FISH was first reported by Pardue and Gall, who utilized radioisotopes to label nucleic acids [21], marking a new era in molecular cytogenetics. Genomic in situ hybridization (GISH), Oligo-FISH, and BAA-FISH have emerged as derivatives of different probe types. D’Hont et al. utilized repetitive sequences (5S rDNA and 45S rDNA) as probes to reveal the varying chromosome bases of sugarcane *S*. *officinarum*, *S. robustum*, and *S. spontaneum* [22,23]. However, in some hybrid combinations with high parental genome similarities, the effectiveness of GISH is limited. For instance, in sugarcane, *S. officinarum* share highly similar centromeric DNA sequences with *S. spontaneum* [18]. GISH experiments conducted on hybrids of *S. officinarum* and *S. spontaneum* exhibited yellow-green and orange-yellow signals [24]. To accurately distinguish the chromosomes or chromosome fragments of wild species infiltrating hybrids, Huang et al. developed a retrotransposon probe that specifically recognizes the chromosomes or chromosome fragments of *S. spontaneum* through genome analysis of *S. officinarum* and *S. spontaneum* [25]. Thus, FISH combined with the suitable probes will provide a clear and intuitive visualization of the key genetic information in plants. This includes the overall chromosome count, as well as the detection of deletions, translocations, and recombinations of chromosome segments.

In this study, the chromosome karyotypes of *S. spontaneum* hybrids and their parental clones were constructed using Oligo-FISH, and the patterns of chromosome inheritance in *S. spontaneum* were analyzed. Our results provide a new clue regarding in the chromosome inheritance of *S. spontaneum* hybridization and lay an important theoretical foundation for understanding the wide distribution of chromosome numbers of *S. spontaneum* in nature.

## 2. Results

### 2.1. Tetra-Primer ARMS PCR Identification of Real or Fake Hybrids of Spontaneum

**Figure 1:**
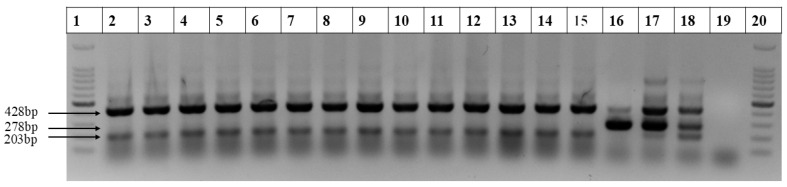
Four parent samples of *S. spontaneum* and their hybrid offspring were analyzed using four-primer ARMS PCR products subjected to agarose gel electrophoresis. Lane 1: 100 bp DNA ladder. Lanes 2–15: 2017-22, 82-1, 2017-41, Yunnan8, A1, A2, A33-1, A18, AA-4, AA-6, AA-10, C1, and C2. Lanes 16–20: Badila, YZ05-51, XTT22, H_2_O, and another 100 bp DNA ladder marker.

To ensure that true hybrid offspring were obtained from *S. spontaneum* hybridization, DNA was extracted from the both parental materials and their hybrid offspring. Tetra-primer ARMS PCR specific primers were employed to authenticate the true *S. spontaneum* offspring [24,26]. Agarose gel electrophoresis results revealed a 428 bp common band for both *S. officinarum* and *S. spontaneum*. Additionally, a distinct 278 bp band was observed in authentic *S. officinarum*, while a unique 203 bp band was found in *S. spontaneum* (Figure 1). The PCR products from the hybrid offspring were detected at a *S. spontaneum*-specific band at 203 bp, and in the absence of the 278 bp band. These results demonstrate that the offspring materials were the true *S. spontaneum* progenies.

### 2.2. Karyotype Construction of S. spontaneum Parents

**Figure 2:**
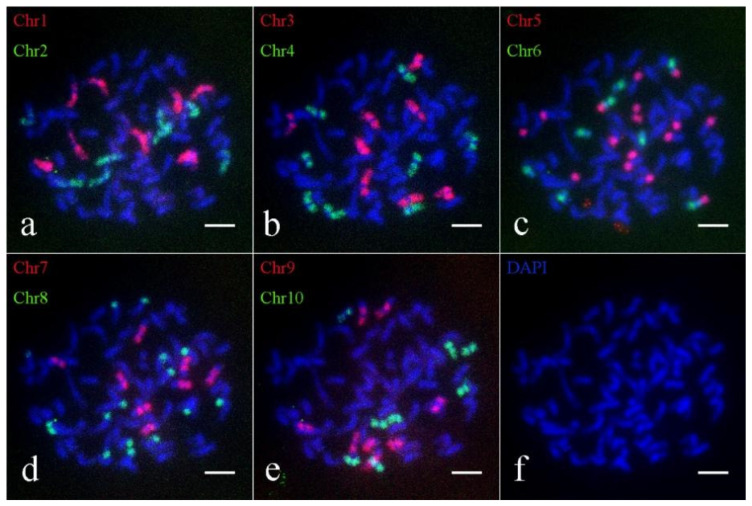
Oligo-FISH results of *S. spontaneum* 82-1. (**a**) Red marker is chromosome 1 (Chr1), green marker is chromosome 2 (Chr2); (**b**) red marker for chromosome 3 (Chr3), green marker for chromosome 4 (Chr4); (**c**) red marker for chromosome 5 (Chr5), green marker for chromosome 6 (Chr6); (**d**) red marker is chromosome 7 (Chr7), green marker is chromosome 8 (Chr8); (**e**) red marker for chromosome 9 (Chr9), green marker for chromosome 10 (Chr10); (**f**) DAPI; the scale is 5 μm.

To thoroughly investigate the chromosome inheritance of *S. spontaneum* materials during hybridization, we employed Oligo-FISH to construct the karyotype of the parental clones *S. spontaneum* Yunnan2017-22 and *S. spontaneum* Yunnan82-1. The Oligo-FISH analysis revealed that the hybrid parent *S. spontaneum* Yunnan82-1 contains 64 chromosomes. Specifically, probes for Chr1, Chr2, Chr3, Chr4, Chr6, Chr7, Chr9, and Chr10 exhibited 8 bright signals, while each Chr5 and Chr8 produced 16 bright signals (Figure 2). Crucially, observations aligned with the previously reported evolution of *S. spontaneum* from *x* = 10 to *x* = 8, involving the breakage and translocation of chromosomes 5 and 8. Chromosome 5 was broken and fused to chromosomes 6 and 7, and chromosome 8 was broken and fused to chromosomes 2 and 9 (Figure 2c,d). These results enabled the drawing of the karyotype pattern for *S. spontaneum* 82-1 (Figure S1A), confirming a chromosome number of 2*n* = 8*x* = 64.

**Figure 3:**
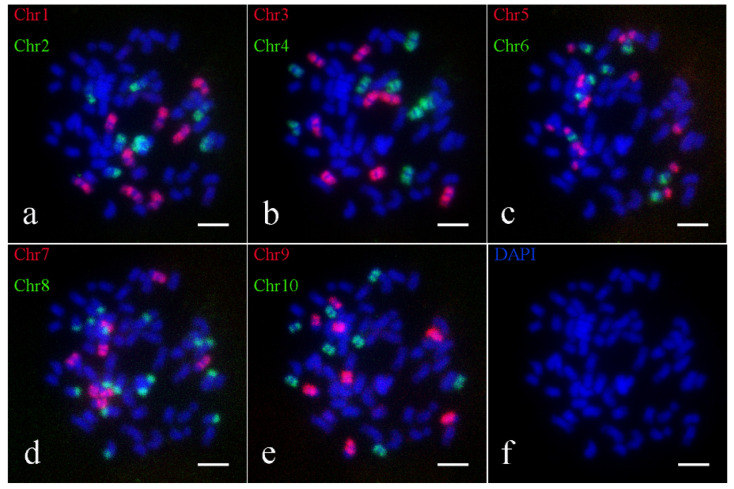
Oligo-FISH results of parent *S. spontaneum* 2017-22. (**a**) red marker is chromosome 1 (Chr1), green marker is chromosome 2 (Chr2); (**b**) red marker for chromosome 3 (Chr3), green marker for chromosome 4 (Chr4); (**c**) red marker for chromosome 5 (Chr5), green marker for chromosome 6 (Chr6); (**d**) red marker is chromosome 7 (Chr7), green marker is chromosome 8 (Chr8); (**e**) red marker for chromosome 9 (Chr9), green marker for chromosome 10 (Chr10); (**f**) DAPI; the scale is 5 μm.

Similarly, Oligo-FISH results also indicated that the presence of 64 chromosomes in the hybrid parent *S. spontaneum* Yunnan2017-22. The probe signals matched those found in *S. spontaneum* Yunnan82-1, with 8 bright signals observed for Chr1, Chr2, Chr3, Chr4, Chr6, Chr7, Chr9 and Chr10, and 16 bright signals for Chr5 and Chr8 (Figure 3). Similar breakage and fusion were also detected in chromosomes 5 and 8. Consequently, the karyotype pattern for *S. spontaneum* 2017-22 was established (Figure S1B), corroborating the chromosome number of 2*n* = 8*x* = 64.

**Figure 4:**
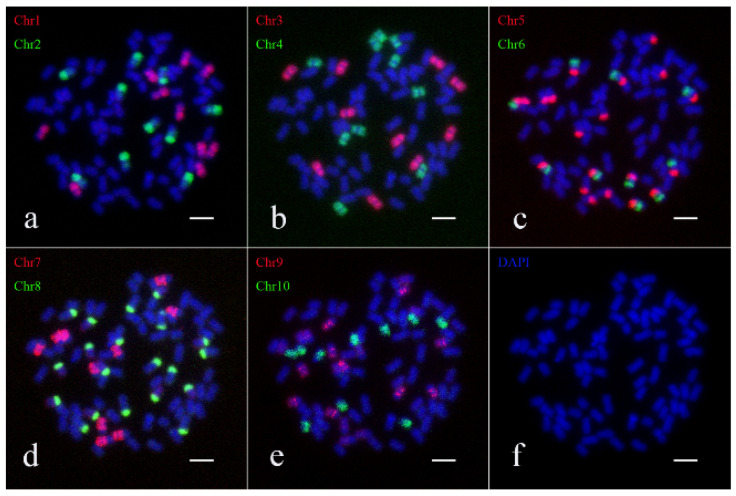
Oligo-FISH results of *S. spontaneum* Yunnan8. (**a**) red marker is chromosome 1 (Chr1), green marker is chromosome 2 (Chr2); (**b**) red marker for chromosome 3 (Chr3), green marker for chromosome 4 (Chr4); (**c**) red marker for chromosome 5 (Chr5), green marker for chromosome 6 (Chr6); (**d**) red marker is chromosome 7 (Chr7), green marker is chromosome 8 (Chr8); (**e**) red marker for chromosome 9 (Chr9), green marker for chromosome 10 (Chr10); (**f**) DAPI; the scale is 5 μm.

Simultaneously, the karyotype of Yunnan8 was established. It was found that *S. spontaneum* Yunnan8 contains 80 chromosomes. The probes for Chr1, Chr2, Chr3, Chr4, Chr6, Chr7, Chr9, and Chr10 each produced 10 bright signals, while Chr5 and Chr8 each produced 20 bright signals (Figure 4). Based on the Oligo-FISH results, the karyotype pattern of Yunnan8 was diagrammed (Figure S2A). In summary, the chromosome karyotype of Yunnan8 is 2*n* = 10*x* = 80.

**Figure 5:**
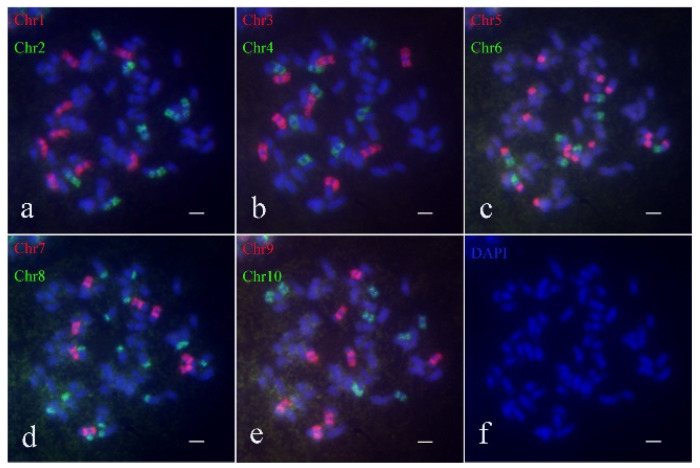
Oligo-FISH results of *S. spontaneum* 2017-41. (**a**) red marker is chromosome 1 (Chr1), green marker is chromosome 2 (Chr2); (**b**) red marker for chromosome 3 (Chr3), green marker for chromosome 4 (Chr4); (**c**) red marker for chromosome 5 (Chr5), green marker for chromosome 6 (Chr6); (**d**) red marker is chromosome 7 (Chr7), green marker is chromosome 8 (Chr8); (**e**) red marker for chromosome 9 (Chr9), green marker for chromosome 10 (Chr10); (**f**) DAPI; the scale is 5 μm.

*S. spontaneum* Yunnan2017-41 contains 64 chromosomes. The probes for Chr1, Chr2, Chr3, Chr4, Chr6, Chr7, Chr9, and Chr10 each produced 8 bright signals, while Chr5 and Chr8 each produced 16 bright signals (Figure 5). The chromosome karyotype pattern of 2017-41 was also diagrammed according to the Oligo-FISH results (Figure S2B). In summary, the karyotype of *S. spontaneum* Yunnan2017-41 is 2*n* = 8*x* = 64.

### 2.3. Karyotype Construction of Hybrid Offspring of Cross A

**Figure 6:**
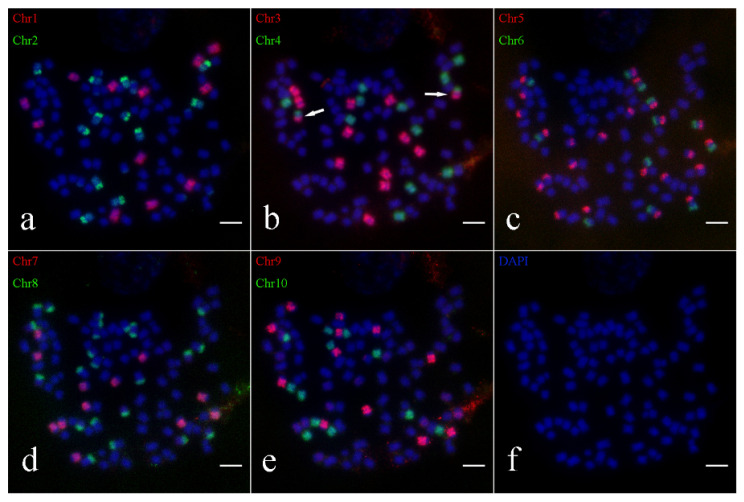
The Oligo-FISH results of progeny material A1. (**a**) red marker is chromosome 1 (Chr1), green marker is chromosome 2 (Chr2); (**b**) red marker for chromosome 3 (Chr3), green marker for chromosome 4 (Chr4); (**c**) red marker for chromosome 5 (Chr5), green marker for chromosome 6 (Chr6); (**d**) red marker is chromosome 7 (Chr7), green marker is chromosome 8 (Chr8); (**e**) red marker for chromosome 9 (Chr9), green marker for chromosome 10 (Chr10); (**f**) DAPI; the scale is 5 μm. In panel (**b**), the white arrow highlights the recombinant chromosome resulting from the recombination of Chr3 and Chr4.

**Figure 7:**
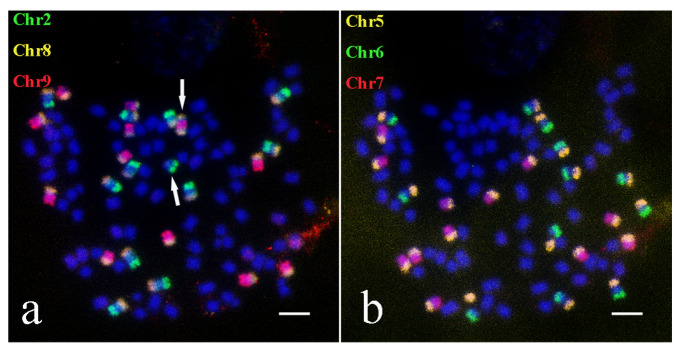
Fusion chromosome results of progeny material A1. (**a**) Chr2 is marked as green, Chr8 is marked as yellow, Chr is marked as red; (**b**) Chr5 marked as yellow, Chr6 marked as green, Chr7 marked as red; the scale is 5 μm. In panel (**a**), the white arrows indicate recombinant chromosomes formed when Chr8 associates with the ends of Chr9 and a fragment of an independent Chr2 chromosome.

We confirmed that the karyotypes of both parents of cross A (Yunnan2017-22 and Yunnan82-1) were 2*n* = 8*x* = 64, with a basic chromosome number of *x* = 8. To investigate chromosome inheritance during hybridization, we constructed the chromosome karyotype of cross A. The analysis revealed that the chromosome number of A1 was 2*n* = 12*x* = 96, indicating a 2*n* + *n* chromosome inheritance. Notably, Chr1, Chr2, Chr6, Chr7, Chr9, and Chr10 each exhibited 12 distinct signals, Chr3 and Chr4 showed 13 distinct signals, and Chr5 and Chr8 showed 24 distinct signals (Figure 6). Oligo-FISH results indicated the presence of two fused chromosomes derived from Chr3 and Chr4 (Figure 6b). According to prior analyses of A1′s parental chromosomes, Chr8 was broken and fused with both Chr2 and Chr9. We overlaid the signals of Chr2, Chr8, and Chr9, and observed the Chr8 signal appeared at both ends of a Chr9 chromosome, yet no Chr8 signal was detected on a Chr2 chromosome. This implied that the Chr8 fragment originally fused to Chr2 was fused with Chr9 (Figure 7a). Similarly, the Chr5 signal appeared at both Chr6 and Chr7 (Figure 7b). Based on the Oligo-FISH results, a chromosome composition map of A1 was created to provide a more intuitive depiction of A1’s chromosome composition (Figure S3).

**Figure 8:**
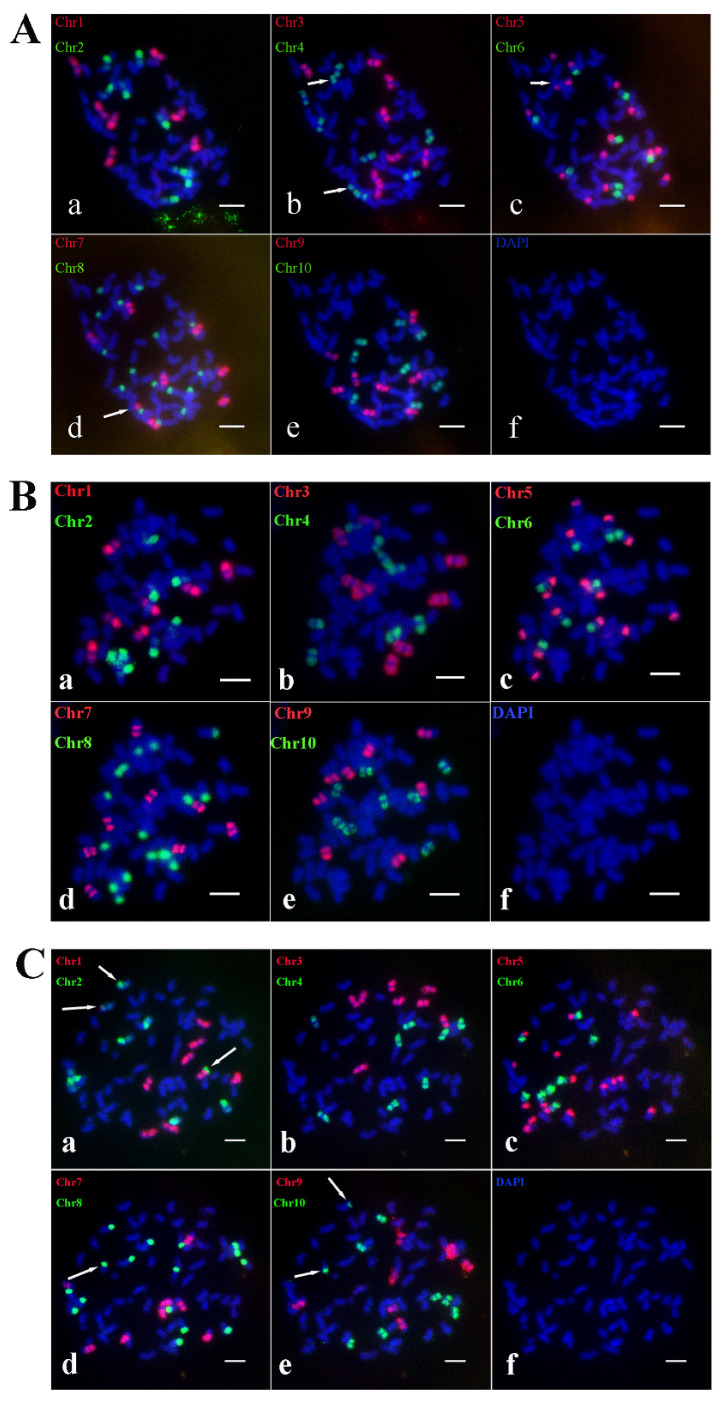
The Oligo-FISH results of progeny material A2, A33-1 and A18. (**A**) The Oligo-FISH results of progeny material A2; (**B**) the Oligo-FISH results of progeny material A33-1; (**C**) the Oligo-FISH results of progeny material A18. (**a**) red marker is chromosome 1 (Chr1), green marker is chromosome 2 (Chr2); (**b**) red marker for chromosome 3 (Chr3), green marker for chromosome 4 (Chr4); (**c**) red marker for chromosome 5 (Chr5), green marker for chromosome 6 (Chr6); (**d**) red marker is chromosome 7 (Chr7), green marker is chromosome 8 (Chr8); (**e**) red marker for chromosome 9 (Chr9), green marker for chromosome 10 (Chr10); (**f**) DAPI; the scale is 5 μm. In panels (**A**(**b**,**d**)), the white arrows indicate that Chr4 formed a recombinant chromosome with Chr7. In panels (**A**(**b**,**c**)), the white arrows show that Chr4 formed a recombinant chromosome with Chr5. The white arrows in panel (**C**(**a**)) indicate that Chr1 formed a recombinant chromosome with Chr2. In panels (**C**(**a**,**e**)), the white arrows highlight that Chr2 formed a recombinant chromosome with Chr10. Finally, the white arrows in panels (**C**(**d**,**e**)) indicate that Chr8 formed a recombinant chromosome with Chr10.

To investigate the presence of this genetic pattern in other offspring from this cross, the karyotypes of offspring A2, A18, and A33-1 from the cross of *S. spontaneum* Yunan2017-22 × *S. spontaneum* Yunan82-1 were constructed. The analysis showed that A2 contains 63 chromosomes. A2 adheres to the traditional n + n inheritance pattern, resulting in a chromosome composition of 2*n* = 8*x* = 63, with a basic chromosome number of *x* = 8, and lost one chromosome 1. Additionally, fused chromosomes involving Chr4 and Chr5 (Figure 8A(b,c)) and Chr4 and Chr7 (Figure 8A(b,d)) were identified. This indicates that Ss4 and Ss6 underwent translocation, with Ss6 exchanging the Chr5-stained segment with Ss4. The chromosome composition pattern of A2 was plotted based on Oligo-FISH results to provide a more intuitive representation of A2’s chromosome structure (Figure S4A).

A33-1 contains 63 chromosomes and follows the traditional n + n inheritance pattern, achieving a chromosome composition of 2*n* = 8*x* = 63, with a basic chromosome number of *x* = 8, and lost one chromosome 1 (Figure 8B). One Ss6 was missing a Chr5-stained segment. The chromosome composition pattern of A33-1 was mapped from the Oligo-FISH results to visually represent its chromosome structure (Figure S4B).

A18 contains 64 chromosomes and follows the traditional n + n inheritance pattern, yielding a chromosome composition of 2*n* = 8*x* = 64, with a basic chromosome number of *x* = 8. Oligo-FISH results revealed 8 signals in Chr3, Chr4, Chr6, Chr7, and Chr9; 9 signals in Chr1, Chr2, and Chr10; and 16 signals in Chr5 and Chr8 (Figure 8C). Among these, Chr1 and Chr2 were found to have undergone crossing-over (Figure 8C(a)), while Chr2 and Chr10, as well as Chr8 and Chr10, recombined to form new chromosomes (Figure 8C(a,e); Figure 8C(d,e)). The fracture in Chr10 disrupted the fusion between Chr2 and Chr8. The karyotype pattern of A18 was plotted to provide a clear visualization of its chromosomal arrangement (Figure S4C).

### 2.4. Karyotype Construction of A1 Selfing Progeny

Due to the special chromosome inheritance pattern of A1, we wanted to explore whether this pattern would occur stably. Therefore, we used the same method to analyze the chromosome composition of offspring resulting from A1 self-fertilization. Unfortunately, the chromosome composition of these selfed offspring remains largely consistent with that of A1, with a karyotype of 2*n* = 12*x* = 94 to 96, indicating only the loss of some chromosomes or some recombined chromosome, without further chromosomal doubling.

**Figure 9:**
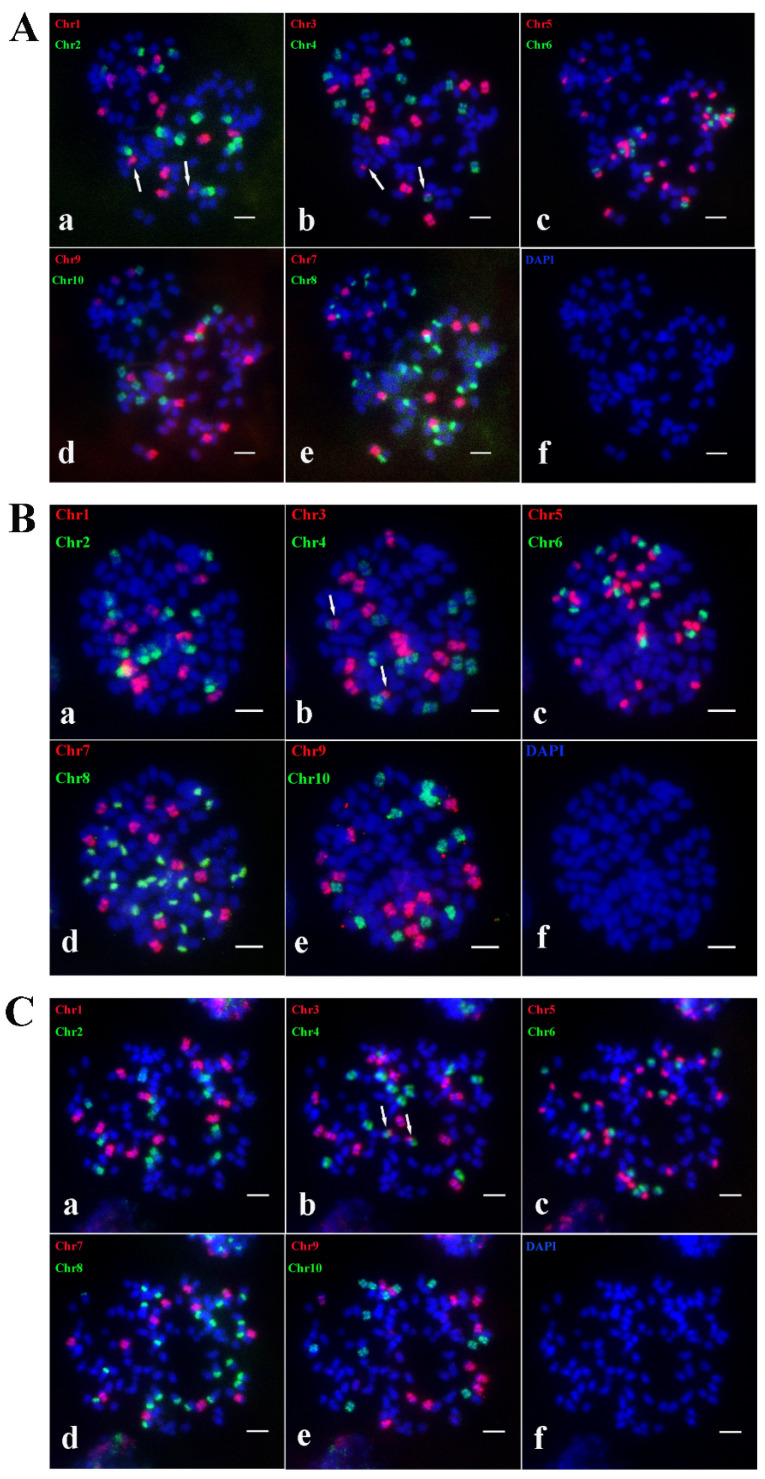
The Oligo-FISH results of progeny material AA-4, AA-6 and AA-10. (**A**) The Oligo-FISH results of progeny material AA-4; (**B**) the Oligo-FISH results of progeny material AA-6; (**C**) the Oligo-FISH results of progeny material AA-10. (**a**) red marker is chromosome 1 (Chr1), green marker is chromosome 2 (Chr2); (**b**) red marker for chromosome 3 (Chr3), green marker for chromosome 4 (Chr4); (**c**) red marker for chromosome 5 (Chr5), green marker for chromosome 6 (Chr6); (**d**) red marker is chromosome 7 (Chr7), green marker is chromosome 8 (Chr8); (**e**) red marker for chromosome 9 (Chr9), green marker for chromosome 10 (Chr10); (**f**) DAPI; the scale is 5 μm. The white arrows in panels (**A**(**a**,**b**)) illustrate recombinant chromosomes formed between Chr1 and Chr3, with a small fragment of Chr1 combining with Chr3 and Chr4 to create another recombinant chromosome. Similarly, in panels (**B**(**b**)) and (**C**(**b**)), the white arrows indicate recombinant chromosomes formed between Chr1 and Chr3.

AA-4 contains 94 chromosomes and follows the traditional n + n inheritance pattern, achieving a composition of 2*n* = 12*x* = 94, *x* = 8. Oligo-FISH results revealed 12 signals in Chr1, Chr2, Chr3, Chr4, Chr6, Chr7, and Chr10 of AA-4, 24 signals in Chr5, 11 signals in Chr9, and 23 signals in Chr8 (Figure 9A). Two small fragments of Chr3 were recombined with Chr1 and Chr4, respectively (Figure 9A(a,b)). To visualize AA-4’s chromosome composition more clearly, we plotted its karyotype pattern (Figure S5A).

AA-6 contains 96 chromosomes and adheres to the traditional n + n inheritance pattern, resulting in a composition of 2*n* = 12*x* = 96, *x* = 8. Oligo-FISH results showed 12 signals in Chr1, Chr2, Chr6, Chr7, Chr9, and Chr10 of AA-6, 13 signals in Chr3 and Chr4, 24 signals in Chr5, and 25 signals in Chr8 (Figure 9B). Two recombinant chromosomes involving Chr3 and Chr4 were identified (Figure 9B(b)). The karyotype pattern of AA-6 was plotted for a more intuitive display of its chromosome composition (Figure S5B).

AA-10 contains 94 chromosomes, and maintains the traditional n + n inheritance mechanism, resulting in a composition of 2*n* = 12*x* = 94, *x* = 8. Oligo-FISH analysis showed 12 signals in Chr1, Chr2, Chr6, Chr7, Chr9, and Chr10 of AA-10, 14 signals in Chr3, 13 signals in Chr4, 24 signals in Chr5, and 22 signals in Chr8 (Figure 9C). Recombinant chromosomes from Chr3 and Chr4 were observed (Figure 9C(b)). The chromosome karyotype pattern of AA-10 was mapped to provide a clear overview of its chromosomal configuration (Figure S5C).

### 2.5. Karyotype Construction of Progenies of Cross C

**Figure 10:**
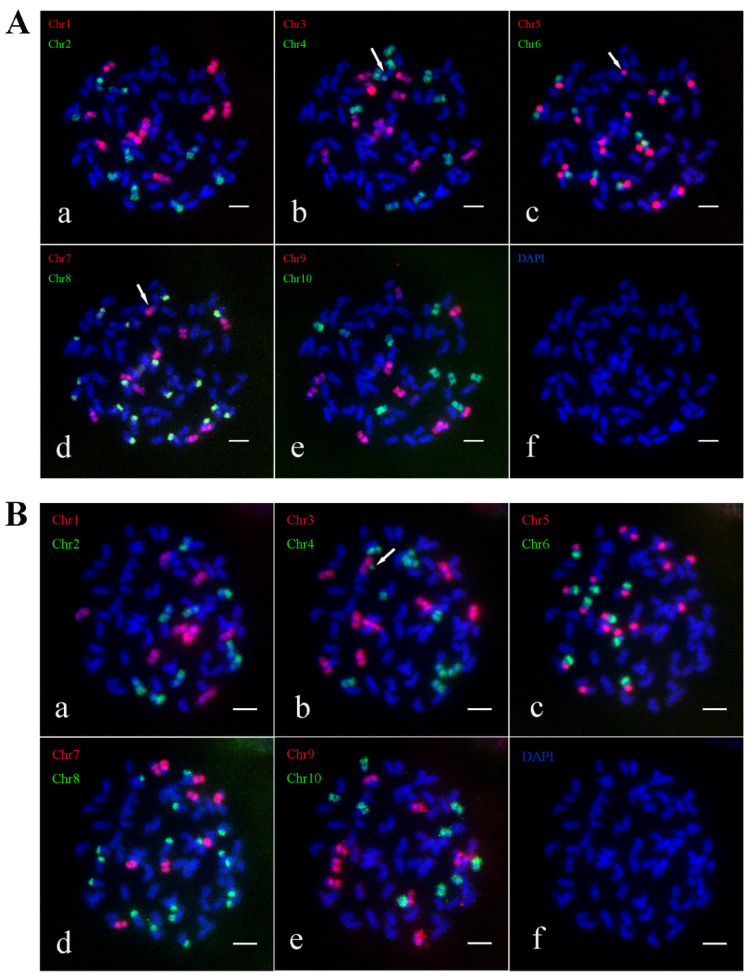
The Oligo-FISH results of progeny material C1 and C2. (**A**) The Oligo-FISH results of progeny material C1; (**B**) the Oligo-FISH results of progeny material C2. (**a**) red marker is chromosome 1 (Chr1), green marker is chromosome 2 (Chr2); (**b**) red marker for chromosome 3 (Chr3), green marker for chromosome 4 (Chr4); (**c**) red marker for chromosome 5 (Chr5), green marker for chromosome 6 (Chr6); (**d**) red marker is chromosome 7 (Chr7), green marker is chromosome 8 (Chr8); (**e**) red marker for chromosome 9 (Chr9), green marker for chromosome 10 (Chr10); (**f**) DAPI; the scale is 5 μm. The white arrows in panels (**A**(**b**–**d**)) highlight recombinant chromosomes formed by Chr4, Chr5, and Chr7. The white arrow in panel (**B**(**b**)) points to a distinct segment of the Chr4 chromosome.

Chromosome identification for clones C1 and C2 of the cross C was conducted using Oligo-FISH. The analysis revealed that C1 contains 72 chromosomes, adhering to the traditional *n* + *n* inheritance pattern, resulting in a chromosome composition of 2*n* = 9*x* = 72, *x* = 8. Oligo-FISH results showed 9 signals in Chr1, Chr2, Chr3, Chr6, Chr7, Chr9, and Chr10; 18 signals in Chr5 and Chr8; and 10 signals in Chr4 (Figure 10A). Notably, a recombinant chromosome involving Chr4, Chr5, and Chr7 was identified (Figure 10A(b–d). The chromosome karyotype pattern of C1 was mapped (Figure S6A).

For C2, the Oligo-FISH analysis indicated 9 signals in Chr2, Chr3, Chr6, Chr7, Chr9, and Chr10; 18 signals in Chr5 and Chr8; 7 signals in Chr1; and 10 signals in Chr4 (Figure 10B). The karyotype pattern of C2 was plotted (Figure S6B).

## 3. Discussion

Sugarcane cultivars exhibit complex allopolyploid genomes (2*n* = 100–140) with frequent aneuploidy. *S. officinarum* (2*n* = 8*x* = 80) and *S. spontaneum* are important parents in sugarcane breeding. The latter displays three fundamental chromosomal types and diverse ploidy levels that generate phenotypic variability. The 2*n* + *n* chromosomal inheritance pattern was first observed in noblizition between *S. officinarum* and *S. spontaneum*, enhancing sugar-related traits by transmitting intact *S. officinarum* chromosomes. However, the mechanism of chromosomal doubling was diverse. Four principal mechanisms drive 2*n* gamete formation: First Division Restitution (FDR), Second Division Restitution (SDR), Post-Meiotic Restitution (PMR), and Indeterminate Meiotic Restitution (IMR) [27,28,29]. FDR preserves homologous chromosome pairs by omitting meiotic I [30], while SDR involves sister chromatid non-disjunction during meiosis II [29]. PMR arises from post-meiotic mitotic duplication [31], and IMR exhibits hybrid FDR/SDR characteristics, though rarely reported except in *Lilium* spp. [32,33,34].

Sugarcane 2*n* gametes commonly occur when *S. officinarum* is crossed with *S. spontaneum*. Intriguingly, our study revealed the unexpected emergence of the 2*n* + *n* inheritance in intraspecific *S. spontaneum* crosses. For instance, crossing *S. spontaneum* 2017-22 (2*n* = 8*x* = 64, *x* = 8) with Yunnan *S. spontaneum* 82-1 (2*n* = 8*x* = 64, *x* = 8) produced rare progeny karyotypes, including A1 (2*n* = 12*x* = 96, *x* = 8) and A2 (2*n* = 8*x* = 64, *x* = 8), suggesting a 2*n* + n inheritance pattern emerged in the interspecific hybridization of *S. spontaneum*. However, A1 karyotypes were exceptionally rare, suggesting stochastic formation events and a lack of molecular evidence to clarify their mechanisms. The formation of 2*n* gametes, facilitated by natural flowering and hybridization in *S. spontaneum*, drives karyotype diversification over evolutionary timescales, contributing to chromosomal diversity distinct from parental lineages. These result in karyotype variations in offspring, differing from parental karyotypes, contributing to the chromosomal diversity of *S. spontaneum*.

Hexaploid wheat (*Triticum aestivum* L.) evolved via distant hybridization and chromosomal doubling among *Triticum* and Aegilops genera [35,36]. Sugarcane breeding leverages hybridization between materials of varying ploidy to generate novel karyotypes [37]. For example, crosses between *S. officinarum* (2*n* = 8*x* = 80, *x* = 10) and *S. spontaneum* (2*n* = 8*x* = 64, *x* = 8) yield progeny with enhanced stress resistance and novel karyotypes [38,39,40]. Oligo-FISH analysis of progeny from *S. spontaneum* 2017-41 (2*n* = 8*x* = 64, *x* = 8) × Yunnan *S. spontaneum* 8 (2*n* = 10*x* = 80, *x* = 8) confirmed typical *n* + *n* chromosomal inheritance, producing a 2*n* = 9*x* = 72, *x* = 8 karyotype. These findings suggest that *S. spontaneum* employs a unique 2*n* + *n* inheritance mechanism to generate karyotypic diversity in natural populations, with extensive chromosomal numerical variation observed across its wild habitats.

Chromosomal translocation enhances the genetic diversity of crops, serving as a critical driver of biological evolution. In agronomic breeding programs, it facilitates the acquisition of novel gene combinations, which can significantly augment disease resistance, yield, and quality of crops [41,42,43]. Our study observed chromosomal deletions and structural rearrangements in *S. spontaneum*, providing a theoretical framework for understanding its chromosomal polymorphism. However, limitations persist: the molecular basis of intraspecific 2*n* gamete formation remains unresolved due to insufficient cytogenetic data, and current conclusions rely heavily on cytological observations. Future integration of single-cell sequencing, epigenetic profiling, and molecular cytogenetic techniques is essential to elucidate the stabilization mechanisms of chromosomal aberrations and advance research on the genetic variability inherent to *S. spontaneum*.

## 4. Materials and Methods

### 4.1. Plant Material

The experimental materials consisted of four original *S. spontaneum* strains (2017-22, 82-1, 2017-41, Yunnan8), six hybrid offspring (A1, A2, A33-1, A18, C1 and C2), and three self-fertilized offspring of A1 (AA-4, AA-6, AA-10). All materials were provided by the Sugarcane Research Institute of the Yunnan Academy of Agricultural Sciences and are preserved in the germplasm nursery of Fujian Agriculture and Forestry University. The pedigree of all plant materials is shown in Table 1.

### 4.2. Experimental Method

#### 4.2.1. Tetra-Primer ARMS PCR Procedure

Genomic DNA was extracted following the instructions provided with the Polysaccharide Polyphenol Plant Genome DNA Extraction Kit (DP360, TIANGEN, Beijing, China). The tetra-primer ARMS PCR was designed based on nrDNA-ITS. The PCR procedure was carried out using the primers FO1, RO1, FI1, and RI1 (FO1: GTTTTTGAACGCAAGTTGCGCCCGAGGC; RO1: AATTCGGGCGACGAA-GCCACCCGATTCT; FI1: GCCGGCGCATCGGCCCTAAGGACCTAT; RI1: GAGCGGCTATGCGCTGCGGTGCTTCT). The reaction system is outlined in Table 2. The thermal cycling conditions were as follows: initial denaturation at 95 °C for 5 min, followed by 6 cycles of 95 °C for 30 s, 78 °C for 20 s (with a 1 °C decrease per cycle), and 72 °C for 20 s. This was followed by 24 cycles of 95 °C for 30 s, 71 °C for 10 s, and 72 °C for 10 s, with a final extension at 72 °C for 5 min. PCR products were analyzed by 1.5% agarose gel electrophoresis [26].

#### 4.2.2. Root Tip Culture and Preparation of Metaphase Chromosome Slides

Sugarcane root tips with vigorous growth were obtained via hydroponics or field cultivation. The meristematic regions of root tips, which exhibit more metaphase chromosomes, were excised and placed in a pre-mixed enzyme solution containing cellulase and pectinase (mixed in a volume ratio of 2:1:1:1 with 1% pectinase Y23, 2% pectinase, 2% RS, and 4% cellulase Onozuka R-10). These were then digested in a precision hybridization chamber at 37 °C for 4 h. Post digestion, root tips were transformed into cell suspension using Carnoy’s fixative, and the suspension was dropped onto slides. Metaphase chromosomes with optimal morphology were observed under a 40× Carl Zeiss microscope (Carl Zeiss, Jena, Germany), and the slides were preserved for fluorescence in situ hybridization (FISH).

#### 4.2.3. Fluorescence In Situ Hybridization Steps

A 100 μL pre-mixed solution (prepared by adding 1 μL of 10 μg/mL pepsin to 99 μL of 0.01 N HCl) was used to cover the slides, placed in a humid chamber, and incubated in a 37 °C hybridization chamber for 1 h. They were then gently immersed in water until the coverglass naturally detached, followed by three washes in 2× SSC for 3 min each. A 100 μL solution of 4% paraformaldehyde was added under a fume hood, covered with a coverglass for 1 min, and then followed by 2× SSC elution for 3 min each, repeated three times. Slides were subsequently rinsed in 70%, 90%, and 100% ethanol for 3 min each and then air dried. Subsequently, 100 μL of 70% FD was applied to the dry slide, coverglass was added, and then this was placed on a 70 °C preheated block for 2 min 30 s. After denaturation, coverslips were quickly removed, and samples were sequentially immersed in 75%, 95%, and 100% ethanol pre-cooled at −20 °C for 5 min each, and then allowed to air dry. The hybridization solution was prepared as follows: 5 μL of 100% FD, 2 μL of 50% DS, 1 μL of 20× SSC, 2 μL of Cy3-modified probe, and 2 μL of FAM-modified probe were added to the PCR tube for FISH, using published probes [19]. The mixture was thoroughly homogenized and denatured at 90 °C in a PCR device for 7 min, before being immediately placed in an ice box to prevent renaturation. Denatured hybrid droplets were applied to slides, covered with coverglass, and sealed with sealant in a humid chamber. The chamber was placed in a 37 °C hybridization box for 18–24 h. Slides were then soaked in 2× SSC until the coverglass detached. Subsequent washing was conducted with 2× SSC for 5 min once, 10 min once, followed by two washes with 1× PBS for 5 min each. Slides were air-dried, and then 10 μL DAPI was applied to the cell area on the slide and covered with a coverglass. Observations were made using a Carl Zeiss Scope A1 fluorescence microscope, and images were captured and processed with OCULAR software (OCULAR 2.0.1.496).

## 5. Conclusions

This study elucidates two distinct genetic mechanisms underlying karyotypic diversification in *S. spontaneum*: the novel 2*n* + *n* inheritance and conventional n + n transmission. Crosses between isokaryotypic octoploid parents (2*n* = 8*x* = 64; *x* = 8) generated aneuploid progeny (A1: 2*n* = 12*x* = 96) with meiotically unstable configurations (2*n* = 94–96 in selfed offspring), demonstrating the transient nature of 2*n* gamete-derived karyotypes. Conversely, hybridization of heterokaryotypic parents (8*x* × 10*x*) via *n* + *n* transmission produced stable 9*x* hybrids (2*n* = 72), highlighting ploidy-dependent inheritance outcomes.FISH-based karyotyping revealed that both mechanisms induce chromosomal deletions (e.g., Chr1 in A2/A33-1/AA-4/C2) and interchromosomal recombinations (Chr3/4 in A1; Chr4/5 in A2; Chr10/8 in A18; Chr10/2 in others), expanding the species’ structural genomic diversity. These modifications—spanning numerical ploidy shifts and complex rearrangements—constitute key drivers of *S. spontaneum*’s natural chromosomal polymorphism.

Our findings establish a mechanistic framework for understanding how meiotic irregularities (2*n* gamete formation) and mitotic instability (somatic recombination) synergistically generate standing chromosomal variation in wild populations. This work provides critical insights for exploiting *S. spontaneum*’s genomic plasticity in sugarcane improvement programs, while advancing fundamental knowledge of polyploid genome dynamics in perennial grasses.

## Figures and Tables

**Table 1:** The information of the materials.

Female	Male	Hybrid Offspring Materials
2017-22 (*S. spontaneum*)	*S. spontaneum* 82-1	A1, A2, A33-1, A18
2017-41 (*S. spontaneum*)	Yunnan8 (*S. spontaneum*)	C1, C2
A1	A1	AA-4, AA-6, AA-10

**Table 2:** Tetra-primer ARMS PCR mixtures.

Components	Volume (μL)
ddH_2_O	1.4
2× GC buffer	10.0
dNTP (2.5 Mm each)	2.4
DimethylsµLphoxide	0.8
FO1 (5 μM)	1.6
RO1 (5 μM)	1.2
FI1 (5 μM)	0.4
RI1 (5 μM)	1.6
Template (gDNA; 50 ng/μL)	0.4
Ex Taq (5 U/μL)	0.2
Total volume	20.0

## Data Availability

The datasets supporting the conclusions of this manuscript and materials generated in this study are available from the corresponding author upon request.

## Supplementary Materials

The following supporting information can be downloaded at: https://www.mdpi.com/article/10.3390/ijms26051958/s1.
