# Supplementary material for: Oligo-FISH-Based Analysis of the Mechanisms Underlying Chromosome Number Variation in Saccharum spontaneum
Source: Int J Mol Sci. 2025 Feb 24;26(5):1958. doi: 10.3390/ijms26051958 (PMC11901062; doi:10.3390/ijms26051958)
Supplement: Supplementary file 1 [file ijms-26-01958-s001.zip › ijms-3458096-supplementary.pdf]

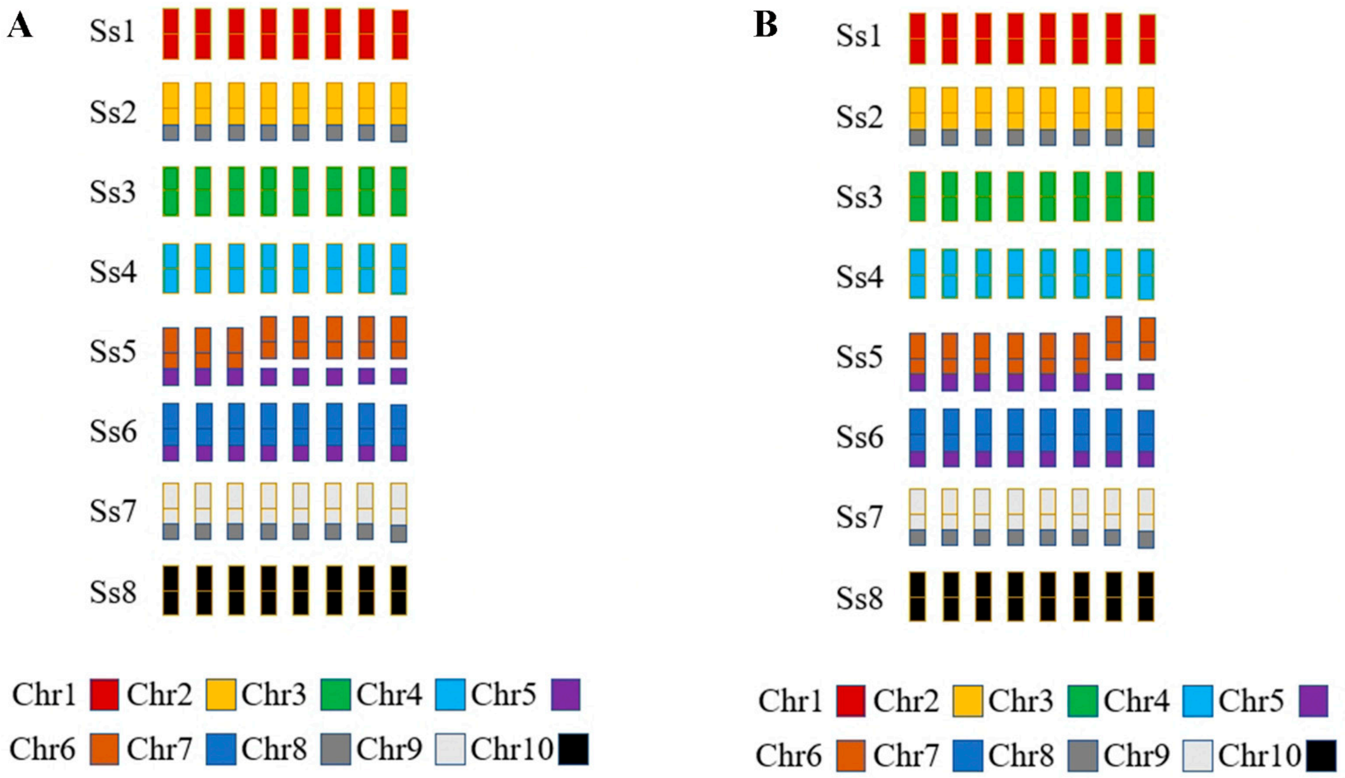

Supplementary Figure S1. Karyotype pattern of parent *S. spontaneum* 82-1 and 2017-22.(A) Karyotype pattern of parent *S. spontaneum* 82-1. (B) Karyotype pattern of parent *S. spontaneum* 2017-22.

Note : Chr is the chromosome number of *S. spontaneum* with x = 10, Ss is the chromosome number of *S. spontaneum* with x = 8.

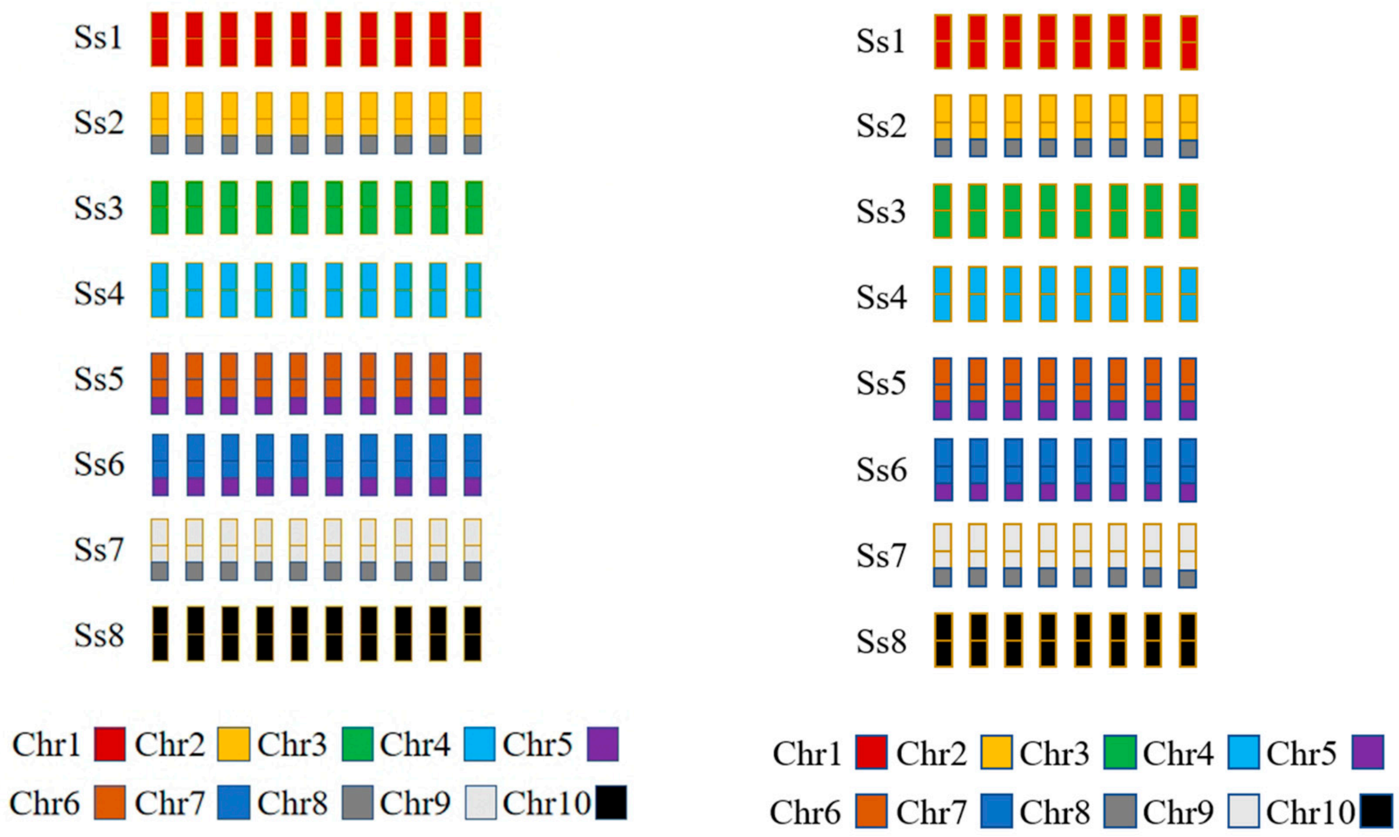

Supplementary Figure S2. Karyotype pattern of parent *S. spontaneum* Yunnan 8 and 2017-41.(A) Karyotype pattern of parent *S. spontaneum* Yunnan 8. (B) Karyotype pattern of parent *S. spontaneum* 2017-41.

Note : Chr is the *S. spontaneum* chromosome number of x = 10, Ss is the *S. spontaneum* chromosome number of x = 8.

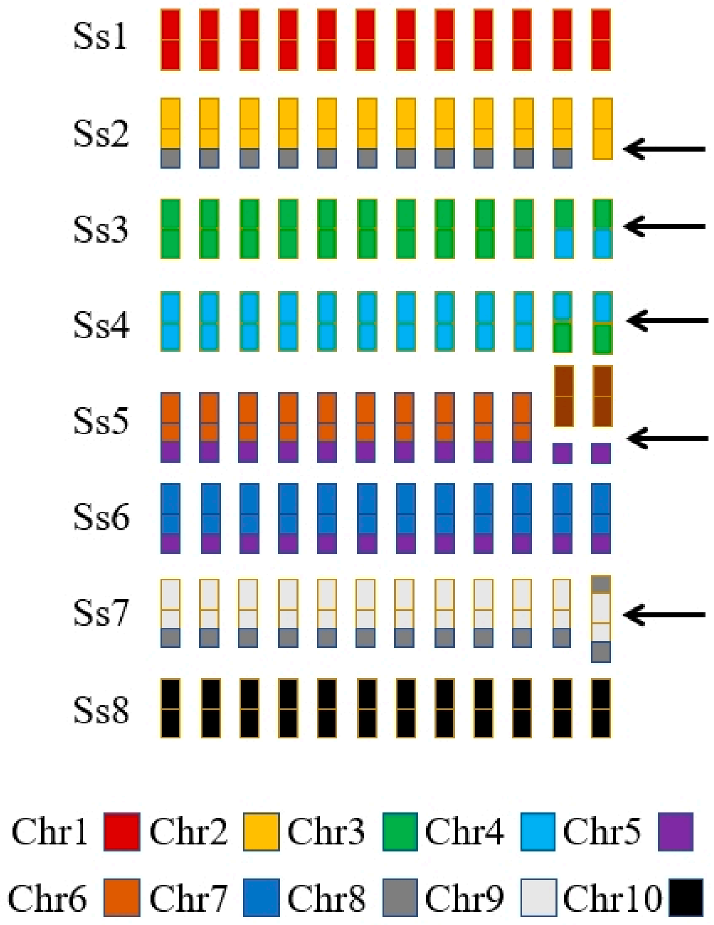

Supplementary Figure S3. Karyotype pattern of *S. spontaneum* A1

Note : Chr is the *S. spontaneum* chromosome number of x = 10, Ss is the *S. spontaneum* chromosome number of x = 8.

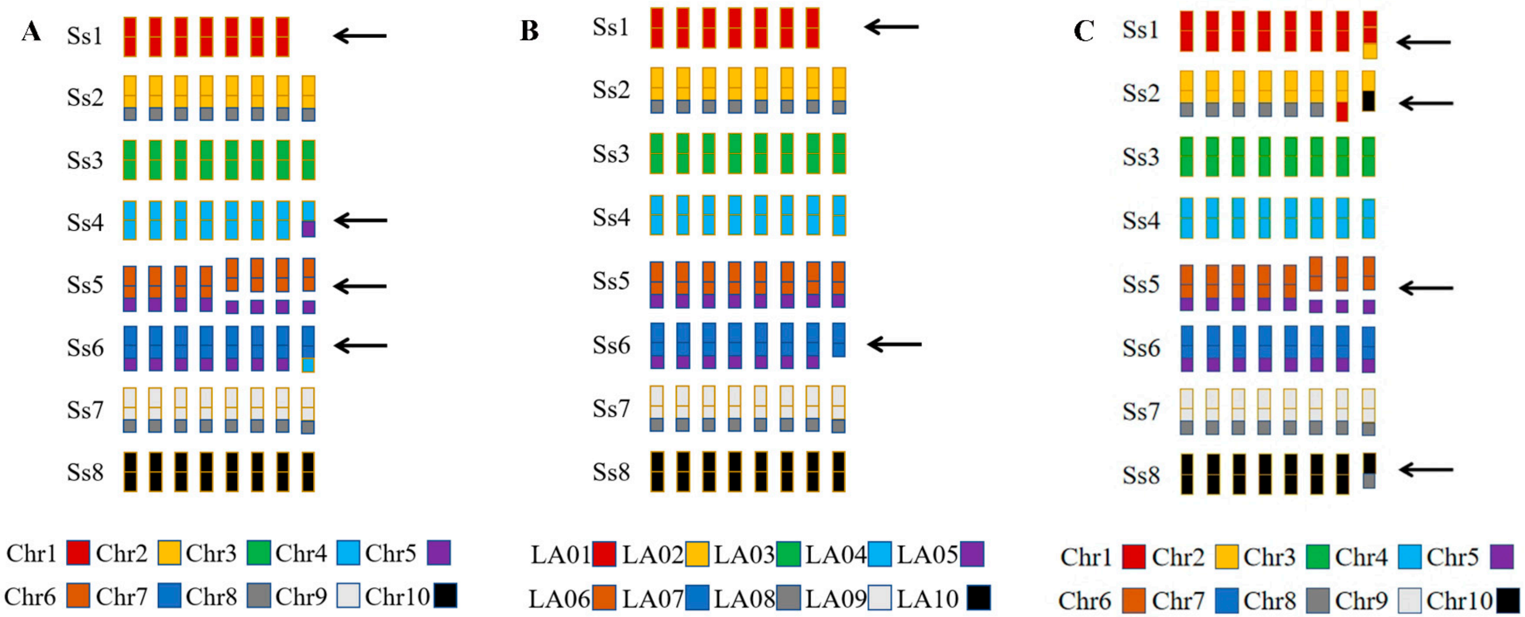

Supplementary Figure S4. Karyotype pattern of *S. spontaneum* A2、A33-1 and A18. (A) Karyotype pattern of *S. spontaneum* A2. (B) Karyotype pattern of *S. spontaneum* A33-1. (C) Karyotype pattern of *S. spontaneum* A18.

Note : Chr is the *S. spontaneum* chromosome number of x = 10, Ss is the *S. spontaneum* chromosome number of x = 8.

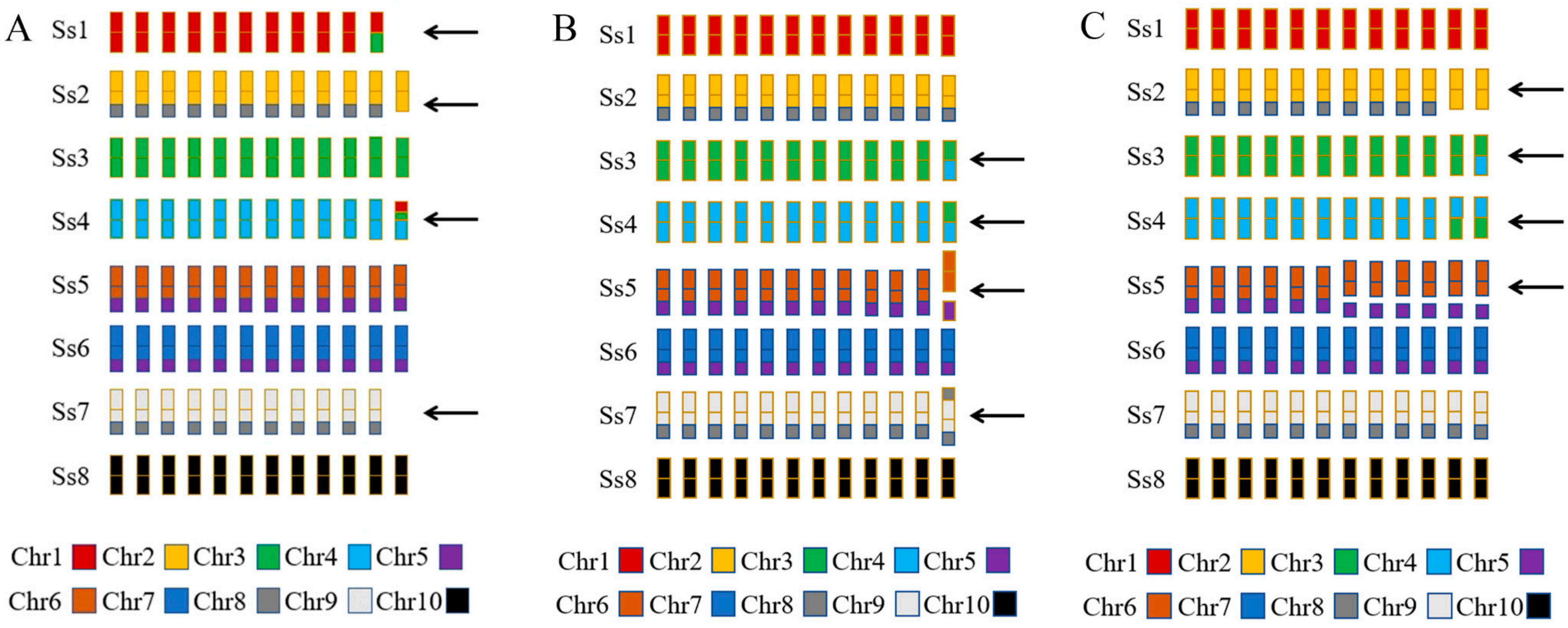

Supplementary Figure S5. Karyotype pattern of *S. spontaneum* AA-4、AA-6 and AA-10.(A) Karyotype pattern of *S. spontaneum* AA-4. (B) Karyotype pattern of *S. spontaneum* AA-6. (C) Karyotype pattern of *S. spontaneum* AA-10.

Note : Chr is the *S. spontaneum* chromosome number of x = 10, Ss is the *S. spontaneum* chromosome number of x = 8.

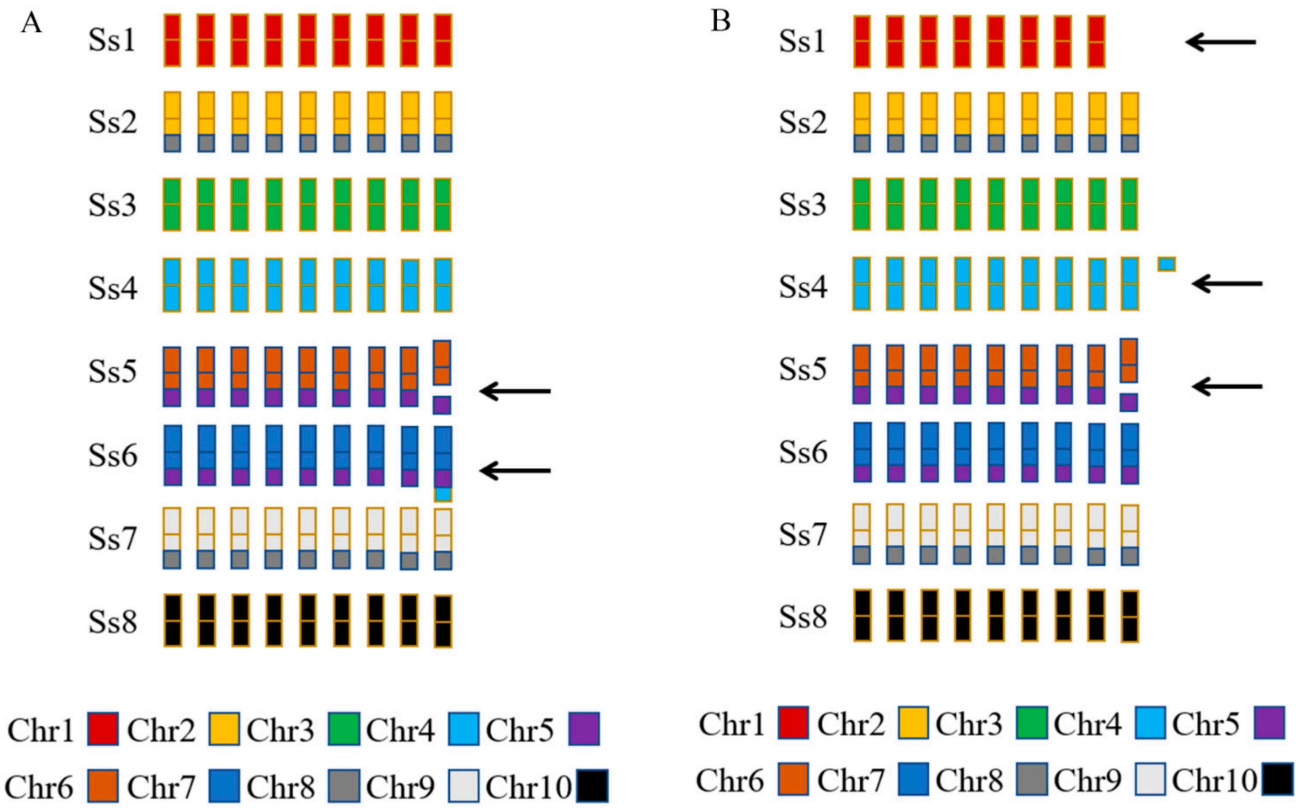

Supplementary Figure S6. Karyotype pattern of *S. spontaneum* C1 and C2.(A) Karyotype pattern of *S. spontaneum* C1. (B) Karyotype pattern of *S. spontaneum* C2.

Note : Chr is the *S. spontaneum* chromosome number of x = 10, Ss is the *S. spontaneum* chromosome number of x = 8.
